# Physiogenomic comparison of human fat loss in response to diets restrictive of carbohydrate or fat

**DOI:** 10.1186/1743-7075-5-4

**Published:** 2008-02-06

**Authors:** Richard L Seip, Jeff S Volek, Andreas Windemuth, Mohan Kocherla, Maria Luz Fernandez, William J Kraemer, Gualberto Ruaño

**Affiliations:** 1 Genomas, Inc., 67 Jefferson St, Hartford, Connecticut, USA; 2 Department of Cardiology, Hartford Hospital, Hartford, Connecticut, USA; 3 Department of Kinesiology, University of Connecticut, Storrs, Connecticut, USA; 4 Department of Nutritional Sciences, University of Connecticut, Storrs, Connecticut, USA

## Abstract

**Background:**

Genetic factors that predict responses to diet may ultimately be used to individualize dietary recommendations. We used physiogenomics to explore associations among polymorphisms in candidate genes and changes in relative body fat (Δ%BF) to low fat and low carbohydrate diets.

**Methods:**

We assessed Δ%BF using dual energy X-ray absorptiometry (DXA) in 93 healthy adults who consumed a low carbohydrate diet (carbohydrate ~12% total energy) (LC diet) and in 70, a low fat diet (fat ~25% total energy) (LF diet). Fifty-three single nucleotide polymorphisms (SNPs) selected from 28 candidate genes involved in food intake, energy homeostasis, and adipocyte regulation were ranked according to probability of association with the change in %BF using multiple linear regression.

**Results:**

Dieting reduced %BF by 3.0 ± 2.6% (absolute units) for LC and 1.9 ± 1.6% for LF (p < 0.01). SNPs in nine genes were significantly associated with Δ%BF, with four significant after correction for multiple statistical testing: rs322695 near the retinoic acid receptor beta (*RARB*) (p < 0.005), rs2838549 in the hepatic phosphofructokinase (*PFKL*), and rs3100722 in the histamine N-methyl transferase (*HNMT*) genes (both p < 0.041) due to LF; and the rs5950584 SNP in the angiotensin receptor Type II (*AGTR2*) gene due to LC (p < 0.021).

**Conclusion:**

Fat loss under LC and LF diet regimes appears to have distinct mechanisms, with *PFKL *and *HNMT *and *RARB *involved in fat restriction; and *AGTR2 *involved in carbohydrate restriction. These discoveries could provide clues to important physiologic mechanisms underlying the Δ%BF to low carbohydrate and low fat diets.

## Introduction

Dietary modification remains a logical and fundamental approach to the treatment of obesity. Achieving success may depend on the diet chosen [1-4] and on innate and genetically inherited metabolic characteristics [5]. Ideally, a weight loss diet regimen should decrease excess adipose tissue mass, including the more important visceral fat, and preserve lean tissue. While weight loss occurs with any dietary strategy that restricts energy intake, it is clear that macronutrient composition has a role in determining adipose tissue and lean body mass responses [6]. In particular, changes in dietary carbohydrate affect substrate metabolism through responses of hormones such as insulin [7] that clearly induce macronutrient-dependent expression of genes [8,9]. Further, there are interindividual differences in metabolic processing of both carbohydrate [10] and fat [11,12] that have been implicated in obesity. In the present study, we compared the genetic variability associated with relative fat loss induced by energy restricted diets that varied in carbohydrate content. Such knowledge may ultimately lead to the development of DNA guided dietary recommendations.

The genes that influence the adipose tissue and lean body (skeletal muscle + bone) mass compartments alone or in response to dietary intervention remain uncharacterized [13,14]. Sorenson et al. [15] examined 42 SNPs in 26 candidate genes hypothesized to modulate obesity but found none to be predictive of the change in body mass index (BMI) induced by dietary restriction of either carbohydrate or fat. Although commonly used, BMI is not a precise surrogate index of adiposity. Its use as a phenotype may make genetic associations with a change in adiposity, difficult to discern.

In order to simultaneously assess relationships between many gene variants and the phenotype of relative body fat, the present study employed physiogenomics [16], a medical application of sensitivity analysis and systems engineering. Sensitivity analysis is the study of the relationship between input and output from a system as determined by system components. Physiogenomics utilizes the genes as the components of the system. The gene variability, measured by single nucleotide polymorphisms (SNPs), is correlated to physiological responses, the output, of a diversely responsive human population. Physiogenomics determines how the SNP frequency varies among individuals similarly responding to the input over the entire range of the response distribution. We have previously utilized physiogenomics to identify genes relevant to dietary weight reduction [5] and drug-induced side effects [17-19].

The present study succeeds a previous physiogenomic study which found that the total body weight loss response to carbohydrate restriction was associated with variants in gastric lipase (*LIPF*), hepatic glycogen synthase (*GYS2*), cholesteryl ester transfer protein (*CETP*) and galanin (*GAL*) genes [5]. Here we extend that study to compare carbohydrate restriction to fat restriction, using the change in relative fat measured by dual energy X ray absorptiometry (DXA), an accurate method to assess percent fat [20,21], as a phenotype. We hypothesized that genes representative of food intake, energy homeostasis, and adipocyte regulation may explain the variability in the change in percent body fat accomplished through restriction of fat or carbohydrate.

## Methods

### Subjects and study design

The subjects included 93 adults who participated in very low carbohydrate (LC) [1,22-25] and 70 who participated in low fat (LF) dietary studies [1,22] designed to examine the effects on weight loss, body composition, and other metabolic responses related to cardiovascular disease in the Human Performance Laboratory at the University of Connecticut (Table 1). The subjects were free of diabetes, cardiovascular, respiratory, gastrointestinal, thyroid and other metabolic disease. They were weight stable (± 2 kg) the month prior to starting the study. Subjects were not allowed to use nutritional supplements (except a daily multi-vitamin/mineral), or take medications to control blood lipids or glucose. The majority of subjects were sedentary and all were instructed to maintain the same level of physical activity throughout the study. The LC diet intervention was 12 weeks for the majority of subjects, but in some cases the duration was shorter (4–6 wk). Before and after LC and LF, body mass was determined in the morning after an overnight fast on a calibrated digital scale with subjects in light clothing and not wearing shoes. All subjects signed an informed consent document approved by the University of Connecticut Institutional Review Board.

**Table 1:** Demographic characteristics of the subjects in the low fat (LF) and low carbohydrate (LC) diet groups.

		***Low Fat***			***Low Carbohydrate***		
**Variable**	**Value**	**Subjects**	**Baseline % Body fat**	**Mean change, % Body Fat [absolute %]**	**Subjects**	**Baseline % Body fat**	**Mean change, % Body Fat [absolute %]**
All	All	70	35.5	-2.02	93	34.6	-2.97
Gender	Female	33	37.6	-1.49	31	38.0	-1.27
	Male	37	33.7	-2.43	62	32.9	-3.80
Age	<20	8	34.4	-3.00	7	34.8	-4.70
	20–30	26	32.2	-1.56	36	33.4	-2.36
	30–40	17	39.6	-2.32	20	37.2	-2.81
	40–50	13	33.9	-2.60	21	35.0	-2.72
	50–60	7	41.6	-0.85	6	32.6	-4.93
	60–70	0	-	-	3	32.5	-6.00
Heritage	African Amer	4	37.9	-1.63	5	36.7	-2.82
	Asian	1	36.5	-3.08	4	36.2	-3.34
	Caucasian	63	35.4	-2.01	81	34.5	-2.50
	Hispanic	2	32.3	-0.99	3	34.7	-2.16

### Dietary protocols

#### Low carbohydrate (LC)

The LC diet intervention has been described previously [5]. Subjects were free-living with the main goal to restrict carbohydrate (CHO) to a level that induced a small level of ketosis. There were no restrictions on the type of fat from saturated and unsaturated sources or cholesterol levels. Foods commonly consumed were beef (e.g., hamburger, steak), poultry (e.g., chicken, turkey), fish, vegetable oils, various nuts/seeds and peanut butter, moderate amounts of vegetables, salads with low CHO dressing, moderate amounts of cheese, eggs, protein drinks, and water or low CHO diet drinks. To ensure appropriate CHO restriction, subjects monitored their level of ketosis daily using urine reagent strips that produce a relative color change in the presence of one of the primary ketones, acetoacetic acid. Blood ketones were also checked during the diets. On this basis, all subjects were in ketosis for the majority of the experimental period. The actual mean nutrient breakdown of the diets as a percentage of total energy was obtained from at least 15 days of weighed food records across the various studies from which subjects were pooled.

#### Low fat (LF)

The LF diet was designed to provide <10% of total calories from saturated fat and <300 mg cholesterol/day [1]. Foods encouraged included whole grains (breads, cereals, and pastas), fruit and fruit juices, vegetables, vegetable oils, low-fat dairy and lean meat products. Standard diabetic exchange lists were used to foster a macronutrient balance of protein (~20% energy), fat (~25% energy), and carbohydrate (~55% of energy). All subjects received extensive initial verbal and written instructions and weekly follow-up dietetic education. Subjects received thorough instructions for completing detailed weighed food records during baseline and various phases of the diet that were subsequently analyzed using regularly updated nutrient analysis software (NUTRITIONIST PRO™, Version 1.5, First Databank Inc, The Hearst Corporation, San Bruno, CA). The actual mean nutrient breakdown of the diets as a percentage of total energy was obtained from at least 15 days of weighed food records.

### Percent body fat measurement

Whole body composition was assessed using DXA (Prodigy™, Lunar Corporation, Madison, WI). Analyses were performed by the same technician (blinded as to dietary intervention) using commercial software (enCORE version 6.00.270). Percent body fat was calculated as soft tissue fat mass divided by the sum of soft tissue fat mass, soft tissue lean body mass, and bone mineral content. Coefficients of variation for lean body mass, fat mass, and bone mineral content on repeat scans with repositioning on a group of men and women in our laboratory were 0.4, 1.4, and 0.6%, respectively.

### Selection of candidate genes

To identify genes involved in physiological responses to carbohydrate versus fat ingestion, we canvassed physiological pathways and biological processes relevant to the regulation of adiposity, including but not limited to eating behavior, digestion and absorption, hormonal signaling in the prandial and postprandial period, the regulation of fuel distribution and processing, and the control of adipocyte size and proliferation. From these categories, we selected 28 genes to represent three categories: food intake, energy homeostasis, and adipocyte regulation. Table 2 provides summary information relevant to the genes and SNPs. The role for each gene is described below.

**Table 2:** Genes and SNPs analyzed for associations with percent body fat change profiles for LF and LC groups.

**Area**	**Pathway**	**Gene**	**Symbol**	**SNP**	**Type**
*Food Intake*	*Neural*	galanin	GAL	rs694066	intron 1
		leptin receptor	LEPR	rs7602	intron 1 (3' UTR on another gene)
				rs1171276	intron 1 (untranslated)
				rs8179183	exon 12, N656K
		melanocortin 3 receptor	MC3R	rs6024725	~10 kb upstream
		neuropeptide Y	NPY	rs1468271	intron 1
		neuropeptide Y receptor Y5	NPY5R	rs11100494	intron 3
				rs6837793	~9 kb upstream
	*Neural/Gut*	histamine N-methyltransferase	HNMT	rs1801105	exon 4, I105T
				rs12691940	intron 2
	*Gut*	apolipoprotein A-IV	APOA4	rs675	T367S, exon 3
				rs5092	exon 2, T29T
		ghrelin precursor	GHRL	rs26312	~1 kb upstream
		lipase, gastric	LIPF	rs814628	exon 4, A161T
		peptide YY	PYY	rs1058046	exon 2, R72T
				rs231460	~1.8 kb upstream
*Energy Homeostasis*	*Metabolic Enzyme*	glycogen synthase kinase 3 beta	GSK3B	rs4688046	intron 3
				rs334555	intron 1
				rs10934502	intron 2
		glycogen synthase 1 (muscle)	GYS1	rs2287754	5' UTR
		glycogen synthase 2 (liver)	GYS2	rs1478290	upstream, ~3.5 Kb
				rs2306179	intron 5
				rs10505873	intron 3
		phosphofructokinase, liver	PFKL	rs2838549	intron 8
		phosphofructokinase, muscle	PFKM	rs2269935	~700 bp upstream
		pyruvate kinase, liver and RBC	PKLR	rs3762272	intron 2
		pyruvate kinase, muscle	PKM2	rs2856929	intron 7 (MT)
	*Nuclear Signaling*	retinoic acid receptor, alpha	RARA	rs4890109	intron 3
				rs9904270	~7.5 kb upstream
		retinoic acid receptor, beta	RARB	rs2033447	intron 2 (MT)
				rs1290443	intron 3 (MT)
				rs322695	~100 kb upstream
		retinoic acid receptor, gamma	RARG	rs10082776	intron 2 (untranslated)
		retinoid X receptor, alpha	RXRA	rs4917348	~100 kbp upstream
				rs3750546	~100 kb upstream
				rs3118536	intron 3
		retinoid X receptor, gamma	RXRG	rs157864	intron 4
*Adipocyte Regulation*	*Adipokine-related*	adiponectin receptor 2	ADIPOR2	rs2058112	intron 1 (untranslated?)
				rs7975375	intron 1 (untranslated?)
		angiotensin II receptor, type 1	AGTR1	rs12695902	intron 3
				rs931490	intron 2 (untranslated?), (MT)
		angiotensin II receptor, type 2	AGTR2	rs5950584	~4.5 kb upstream
		resistin	RETN	rs3219177	intron 1
	*Lipid Metabolic*	apolipoprotein E	APOE	rs7412	exon 3, C176R, *2–>*3
				rs429358	exon 3, R130C, *4–>*3
				rs405509	~200 bp upstream
				rs439401	~1.5 kbp downstream
				rs446037	~1.5 kbp upstream
		cholesteryl ester transfer protein, plasma	CETP	rs5883	exon 9, F287F
				rs1532624	intron 7
				rs3764261	~2.6 kb upstream
				rs5880	nonsynonymous, P390A

#### Food intake

The *GAL *and *GHRL *genes, expressed in the hypothalamus [26] and stomach [27], respectively, encode for the orexigens galanin and ghrelin, respectively [26,27]. *HNMT *encodes for histamine N-methyl transferase, which inactivates histamine and is widely expressed in the stomach, thymus, lung, spleen, kidney, and particularly the brain [28]. *LIPF *encodes for gastric lipase, which hydrolyzes triglycerides, freeing fatty acids for intestinal uptake [29]. The *PYY *gene encodes for peptide YY, a gut endocrine factor that circulates after meals [30]. *NPY *encodes for neuropeptide Y, an abundant brain and autonomic neurotransmitter [31] and potent orexigen [32]. *NPY5R *encodes for one of five neuropeptide Y receptors studied in relation to energy balance [33]. The *LEPR *gene encodes for the leptin receptor, believed critical in the central regulation of energy homeostasis [34]. *MC3R *encodes for the melanocortin receptor 3, which regulates energy homeostasis in response to neuropeptides secreted by pro-opiomelanocortin and agouti related peptide releasing neurons [35]. *APOA4 *encodes for apolipoprotein A-IV, is expressed by intestinal cells in response to fat absorption [36], and has a hypothesized, centrally-mediated role in the regulation of energy intake [36].

#### Energy homeostasis

*GSK3B *encodes for glycogen synthase kinase 3 β-9, which inactivates glycogen synthase by phosphorylation [37]. Glycogen synthase (*GYS*) catalyzes the rate-limiting step in glycogen synthesis. *GYS1 *and *GYS2 *are expressed liver and muscle, respectively. The *PFKL *and *PFKM *genes encode the liver [38] and muscle [39] isoforms of phosphofructokinase, respectively. Phosphofructokinase is the key regulatory enzyme for glycolysis. *PKLR *and *PKM2 *encode for liver and muscle isoforms of pyruvate kinase, an enzyme of glycolysis and gluconeogenesis [37]. The *RARA*, *RARB*, and *RARG*; and *RXRA *and *RXRG *genes are members of the nuclear receptor superfamily. The *RAR *and *RXR *gene products participate in the regulation of energy balance at a neural level [40] and their expression in hepatic and adipose tissues is diet-responsive [41,42].

#### Adipose regulation

The *ACGT1 *and *AGTR2 *genes express angiotensin II receptors type I and II. Both play roles in adipocyte regulation and cellularity [43]. The *ADIPOR2 *gene encodes adiponectin receptor type I; muscle expression of this gene plays a role in non-oxidative glycolysis [44]. Apolipoprotein E and cholesteryl ester protein, encoded by the *APOE *and *CETP *genes, respectively, have widespread roles in lipid metabolism. Both are expressed in adipocytes [45-47] and subject to nutritional regulation [45,48]. *RSTN *encodes for the adipocyte-secreted factor, resistin, which is increased in obesity [49].

#### Laboratory analysis

Blood samples were collected from an arm vein into tubes for DNA extraction. The DNA was extracted from 8.5 mL of whole blood using the PreAnalytiX PAXgene DNA isolation kit (Qiagen Inc, Valencia, CA). For some earlier participants, neither whole blood nor DNA were available, so DNA from lymphocytes remaining in archived serum samples were amplified using the QiaGen REPLI-g Whole Genome Amplification kit. Genotyping was performed using the Illumina BeadArray™ platform and the GoldenGate™ assay [50,51]. The assay information and observed allele frequencies for the SNPs used in this study are listed in Table 3.

**Table 3:** Assay DNA sequences for the SNPs analyzed.

**SNP**	**Gene**	**Chr**	**mac**	**min**	**maj**	**Freq**	**Sequence Context**
rs694066	GAL	11	20	A	G	0.09	TTCTAAGTCCTCTGCCATGCC [A/G]GGAAAGCCTGGGTGCACCCA
rs7602	LEPR	1	29	A	G	0.16	CTTGGAGAGGCAGATAACGCT [A/G]AAGCAGGCCTCTCATGACCC
rs1171276	APOA4	1	23	A	G	0.13	AGTTTCATGTACATTAAATAT [A/G]AATTTCTTTTGGCTGGAAAT
rs8179183	APOA4	1	31	C	G	0.18	TAATGGAGATACTATGAAAAA [C/G]GAGAAAAATGTCACTTTACT
rs6024725	MC3R	20	31	T	C	0.22	CCTAGAGACATATCTCAGTTA [A/G]GTTTTAGCCTCACCAGTATT
rs1468271	NPY	7	12	A	G	0.06	GACCCTGTAATTTTCAGAAAC [A/G]CACATAGGAGTGGGTGTCTG
rs11100494	NPY5R	4	10	A	C	0.05	CAGAAAGATGTCATCATCCAG [A/C]ATTGCGTCCACACAGTCAAC
rs6837793	NPY5R	4	18	A	G	0.10	ATGAATTGTCACTCAGAAGAA [A/G]CTTAATAGGCATTAATACTA
rs1801105	HNMT	2	14	T	C	0.08	TTTACGTTCTCGAGGTTCGAT [A/G]TCTTGGCTACAAGCTCTAAA
rs12691940	HNMT	2	66	A	G	0.00	AATCAACCAAGTGGAAGAAAG [A/G]ATATCAGAGTCTGAAGACAA
rs675	APOA4	11	32	T	A	0.16	GAGAAAGAGAGCCAGGACAAG [A/T]CTCTCTCCCTCCCTGAGCTG
rs5092	APOA4	11	30	A	G	0.16	CAGTGCTGACCAGGTGGCCAC [A/G]GTGATGTGGGACTACTTCAG
rs26312	GHRL	3	36	A	G	0.15	GCTGTTGCTGCTCTGGCCTCT [A/G]TGAGCCCCGGGAGTCCGCAG
rs814628	LIPF	10	26	A	G	0.14	ATCGACTTCATTGTAAAGAAA [A/G]CTGGACAGAAGCAGCTACAC
rs1058046	PYY	17	78	C	G	0.38	GGAAAAGAGACGGCCCGGACA [C/G]GCTTCTTTCCAAAACGTTCT
rs231460	PYY	17	43	T	C	0.23	TGCTCACCCTAGGATGGAGGG [A/G]GCAGTGGGGGCTGGTTAGGA
rs4688046	GSK3B	3	44	T	C	0.24	TAGTAAACTATTTCTTCCCAT [A/G]GGAGAAGATGGATTCTTTTC
rs334555	GSK3B	3	24	C	G	0.13	AATTATATCTTATTATTAAAA [C/G]TCTACCAACTCAAAGCTTCC
rs10934502	GSK3B	3	40	T	C	0.24	GCTTCCTTATGTAAAATGTAG [A/G]TATTTCTAAAGTAACGCAAT
rs2287754	GYS1	19	25	A	G	0.15	CGGGAAGCTTGCAAGACGCTC [A/G]GCTTCCTATTGCAAGACCGC
rs1478290	GYS2	12	59	T	G	0.26	AATGTGGCTGAAGCCAAAAGC [A/C]TAATGAATGAGGGGAAGCCT
rs2306179	GYS2	12	52	A	G	0.27	TTTCAGTAGGTTTGCAGGGAA [A/G]CCAACTCAAAGCTATATCTG
rs10505873	GYS2	12	67	T	C	0.49	TGCTCAGCCTTCTTCAATGAC [A/G]GTGTTTTGCTATTGTCTCTA
rs2838549	PFKL	21	15	A	G	0.09	GGACACTGGTTCCACCTCCGC [A/G]TGGCTGTACAGTGCTGCCGA
rs2269935	PFKM	12	53	A	C	0.26	CGGCAATTAGACTGGCTAGAG [A/C]CACCTCAGTCAGGCTCTCCC
rs3762272	PKLR	1	13	A	G	0.06	AACAAAGATTCTCCTTTCCTC [A/G]TTCACCACTTTCTTGCTGTT
rs2856929	PKM2	15	39	A	G	0.24	CAGGCTCAGGGTCTAAATTCC [A/G]TATCCTTTCTTCCATACCCT
rs4890109	RARA	17	10	T	G	0.05	GGCTGCTCAGGGCCTCGTCCA [A/C]CCCCAGCCTGACAGAGAGCT
rs9904270	RARA	17	29	T	C	0.15	GCCTTCCCCTTAGAGAAGAGC [A/G]CCTGCCAGACAAGGGAGAAG
rs2033447	RARB	3	43	T	C	0.20	ATGCCGGGTGCTAGAGATACA [A/G]CAGTGAACATGACAAAGTTC
rs1290443	RARB	3	30	A	G	0.15	AGAAGCTCTTTCATGTTGTCA [A/G]TTTTAGAAATCCAAATCATT
rs322695	RARB	3	29	A	G	0.15	CCTGTAGGATTGTGTTCCTCT [A/G]AAACTGTCCCCTAAATTATG
rs2838549	PFKL	21	51	A	G	0.09	GGACACTGGTTCCACCTCCGC [A/G]TGGCTGTACAGTGCTGCCGA
rs157864	RXRG	1	17	T	C	0.10	ATGATATTGAATTAAAGGAAA [A/G]TGAATGGTCTCAGTCAGAGA
rs3118536	RXRA	9	31	A	C	0.14	CTGCAGGTGCACGGTTTCCTG [A/C]TTGCCCAGGTGTCTCTGAGC
rs4917348	RXRA	9	40	A	G	0.21	GGTGGGGTTAGAGGGGATGGT [A/G]CCTGGCAGTGTGCAGCAGAC
rs3750546	RXRA	9	43	A	G	0.20	CCTGAGGATGAAGGGGCGTCC [A/G]TGGCCAGGCAGCAGTGAGAA
rs157864	PYY	1	17	T	C	0.10	ATGATATTGAATTAAAGGAAA [A/G]TGAATGGTCTCAGTCAGAGA
rs2058112	ADIPOR2	12	27	T	C	0.15	TCTTCTTGCCCTACATACTTC [A/G]AAAGCCCTTGGAGAAATCCT
rs7975375	ADIPOR2	12	31	T	C	0.17	CTTTTCACAGGAAAATTTCTT [A/G]GGAGTCTATTGTCACTGTCT
rs12695902	AGTR1	3	16	A	G	0.09	CATCAGGATTATCAGCATTTA [A/G]GCCAGAGTTGCAAATTAAGT
rs931490	AGTR1	3	23	A	G	0.19	GGCGCCCCCTGGACTTCTGCT [A/G]GAATTTAGATTTAAATAGAT
rs5950584	AGTR2	X	8	T	G	0.04	CTATCCTCAAATGCTATATAA [A/C]CCAACTGGTGGAAAAAAATT
rs3219177	RETN	19	31	T	C	0.15	CCAGGGATCAGTGAGGTCTCT [A/G]AGACCCTTGGGGAGCTTGCC
rs7412	APOE	19	18	T	C	0.08	CGGCCTGGTACACTGCCAGGC [A/G]CTTCTGCAGGTCATCGGCAT
rs429358	APOE	19	31	T	C	0.14	GGTACTGCACCAGGCGGCCGC [A/G]CACGTCCTCCATGTCCGCGC
rs405509	APOE	19	79	A	C	0.00	GAGGACACCTCGCCCAGTAAT [A/C]CAGACACCCTCCTCCATTCT
rs439401	APOE	19	59	T	C	0.34	GAGAACTGAGGGGGTGGGAGG [A/G]GAAGAGAGTGCCGGCGGCTC
rs446037	APOE	19	5	A	C	0.02	AGACACAGGTGACCCAACTCC [A/C]ATGGCTGGCCTAGGCCCCTC
rs5883	CETP	16	13	T	C	0.06	AGCTACCTTGGCCAGCGAGTG [A/G]AAGACTCGCTCAGAGAACCA
rs1532624	CETP	16	76	T	G	0.00	TCTGCCCCTTTGGGCTGCAGC [A/C]TCACAAGCTGTGTGGCGTTG
rs3764261	CETP	16	46	T	G	0.27	AGTGAATGAGATAGCAGACAA [A/C]CCAGATGCCTACCGACAGGT
rs5880	CETP	16	15	C	G	0.08	GATATCGTGACTACCGTCCAG [C/G]CCTCCTATTCTAAGAAAAGC

Chr: chromosome location of the gene; mac: the number of minor alleles found; maj: sequence of the most common allele, major; min: sequence of the least common allele, minor; Freq: frequency (0.00–1.00) of the minor allele in the study population.

### Data analysis

All statistical analysis was performed using the R Statistics Language and Environment [52-54]. Covariates were analyzed using multiple linear regression, and selected using the stepwise procedure. The change in relative fat mass, Δ%BF, was calculated as the difference between post-diet and baseline measurements of %BF. To test for association with SNP genotypes, the residual of Δ%BF after covariate analysis was tested using linear regression on the SNP genotypes. SNP genotype was coded quantitatively as a numerical variable indicating the number of minor alleles: 0 for major homozygotes, 1 for heterozygotes, and 2 for minor homozygotes. The F-statistic p-value for the SNP variable was used to evaluate the significance of association. To test the validity of the p-values, we also performed an independent calculation of the p-values using permutation testing. The ranking of the first three SNPs were identical under permutation and F-statistic analyses (data not shown). To account for the multiple testing of 53 SNPs, we calculated adjusted p-values using Benjamini and Hochberg's false discovery rate (FDR) procedure [55-57].

### LOESS representation

We use a locally smoothed function of the SNP frequency as it varies with Δ%BF to visually represent the nature of an association. LOESS (LOcally wEighted Scatter plot Smooth) is a method to smooth data using a locally weighted linear regression [58,59]. At each point in the LOESS curve, a quadratic polynomial is fitted to the data in the vicinity of that point. The data are weighted such that they contribute less if they are further away, according to the tricubic function

$$w_{i}={\left(1-{\left|\frac{x-x_{i}}{d(x)}\right|}^{3}\right)}^{3},$$

where *x *is the abscissa of the point to be estimated, the *x*_*i *_are the data points in the vicinity, and *d*(*x*) is the maximum distance of *x *to the *x*_*i*_.

## Results

### Dietary intake

The intake of total dietary energy at baseline did not differ between groups, nor did absolute amounts of protein, carbohydrate, and fat (Table 4). Relative to total energy intake, the intakes of protein, carbohydrate, and fat energy at baseline for LC and LF, expressed as percent of total energy, were 15.9%, 50.2%, and 32.6; and 16.6%, 47.7%, and 34.9%; respectively.

**Table 4:** Dietary data at baseline and during LF and LC interventions.

			Intake (grams/day)			
Condition	Group	Total Energy Intake (Kcal/d)	Protein	Carbohydrate	Fat	Alcohol
Baseline	LF	2176.3 ± 518.4	87.3 ± 23.0	273.2 ± 65.5	80.9 ± 29.7	3.5 ± 6.9
	LC	2291.8 ± 735.9	94.3 ± 32.2	267.3 ± 80.5	92.1 ± 43.6	4.4 ± 1.1
Diet	LF	1470.0 ± 348.7^‡^	69.2 ± 19.0^‡^	212.1 ± 59.2 ^‡^	37.3 ± 12.6 ^‡^	3.1 ± 4.6
	LC	1705.2 ± 57.1 ^‡†^	114.9 ± 32.8 ^‡§^	53.2 ± 42.6 ^‡§^	111.9 ± 38.7 ^‡§^	1.9 ± 3.3 *

^† ^p < 0.01, LC vs. LF, within condition

^§ ^p < 1e^-10^, LC vs. LF, within condition

* p < 0.05, Baseline vs. Diet, within group

^‡ ^p < 0.0001, Baseline vs. Diet, within group

Dietary intervention decreased mean total energy intake to 1470 kcal in LF and to 1705 kcal/day in LC (p < 0.01), representing mean changes from baseline of -32.5% and -26.5%, respectively. During intervention, carbohydrate intake averaged 212 g/day (57% of total energy) in LF compared to 53 g/day (12% of total energy intake) in LC. Protein intake averaged 94 g/day (19% of total energy) in LF and 115 g/day (28% of total energy) in LC. Fat intake averaged 37 g/day (23% of total energy) in LF and 112 g/day (59% of total energy) in LC. All between-group differences in macronutrient gram intake were significant (p < 1^e-10^, Table 4).

### Body mass and composition

There were no differences in body size or composition between groups at baseline. With diet intervention, the change in total body, fat, and lean masses were significantly greater in LC (Table 5).

**Table 5:** Baseline and change in total body mass and composition for LC and LF.

	Baseline			Change		
	
Group	Total Body Mass	Fat Mass	Lean Mass	Total Body Mass	Fat Mass	Lean Mass
LF	89.5 ± 21.2	32.2 ± 11.2	54.4 ± 13.1	-4.0 ± 2.9	-3.2 ± 2.2	-0.7 ± 1.9
LC	91.0 ± 20.0	31.8 ± 10.3	56.3 ± 12.8	-6.5 ± 4.1^‡^	-4.8 ± 3.0^‡^	-1.4 ± 2.6*

* p < 0.05, LC vs. LF

^‡ ^p < 0.0001, LC vs. LF

**Figure 1:**
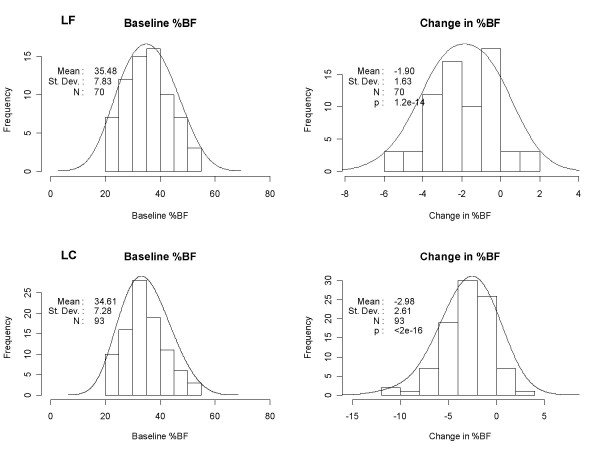
Distribution of baseline and change in percent body fat for LF (top) and LC (bottom) groups. The vertical axes (*Frequency*) indicates the number of patients observed within a given 10% interval up to 60% (baseline, left panels) or within a given 2% or 5% interval (change, right panels) on the horizontal axes. Genotyping was not completed in 3 LF subjects and 7 LC subjects.

Figure 1 depicts the baseline and changes in % body fat profiles in the LF and LC study populations. The distributions are approximately normal and the baseline percent body fat levels for LF (35.4 ± 7.8%) and LC (34.5% ± 7.3%) were not different between groups. Overall LC induced an absolute decrease in % body fat of 2.98 ± 2.62%, reflecting losses of 4.8 kg of fat mass and 1.42 kg of lean mass, compared to a % fat decrease of 1.92 ± 1.63% in LF (p < 0.01), reflecting losses of 3.18 kg for fat mass and 0.70 kg for lean mass. Men outnumbered women in both study groups and had lower baseline % body fat in both groups (33.7 vs. 37.6 LF, and 32.9 vs. 38.0 LC) (Table 1). The change in % body fat (Δ%BF) due to diet was greater for men versus women (Table 5). For physiogenomics analyses, we employed gender as a covariate to account for the difference.

### Physiogenomic associations

Table 6 lists the results of the association tests, comparing LF and LC groups. A single SNP rs322695 in the *RARB *gene was significantly associated with Δ%BF for both the LF and LC interventions (p < 0.0001 and p < 0.0121, respectively). SNPs in the *HNMT *(rs3100722, p < 0.002) and *PFKL *genes (rs2838549, p < 0.002) were significant only for the LF group. Conversely, the rs5950584 SNP in the *AGTR2 *gene was significant only for the LC group (p < 0.0001). The FDR-corrected p values yield an estimate of the false positive rate. The following SNPs, *RARB *rs322695, *HNMT *rs1269140, and *PFKL *rs2838549 and associations in LF, and the *AGTR2 *SNP rs5950584 association in LC are clearly significant after the FDR correction (p < 0.005, p < 0.041, p < 0.041, and p < 0.041, respectively). All remaining genes showed no significant association in either treatment group, and no gene showed significance for both diet treatments after adjusting for multiple tests.

**Table 6:** Significance levels of gene SNPs associated with % body fat change profiles for carbohydrate-restricted (LC) and fat-restricted (LF) diet treatments.

	**Marker**		**P-value**		**Coefficient**		**FDR**	
Area	SNP	Gene	LF	LC	LF	LC	LF	LC

Food Intake	rs1801105	HNMT	0.941	0.028	-0.03	-2.03	0.960	0.368
	rs12691940	HNMT	0.002	0.755	0.89	-0.12	**0.041**	0.974
	rs1468271	NPY	0.049	0.181	-0.89	-0.90	0.321	0.974
Energy Homeostasis	rs1478290	GYS2	0.038	0.009	-0.60	-0.86	0.321	0.209
	rs2838549	PFKL	0.002	0.902	1.37	-0.10	**0.041**	0.974
	rs3762272	PKLR	0.048	0.282	-2.24	-0.60	0.321	0.974
	rs322695	RARB	0.0001	0.012	1.29	1.03	**0.005**	0.209
Adipocyte Regulation	rs2058112	ADIPOR2	0.044	0.141	-0.89	-0.78	0.321	0.974
	rs5950584	AGTR2	0.507	0.0001	-0.77	-3.31	0.732	**0.021**
	rs439401	APOE	0.036	0.691	-0.74	-0.15	0.321	0.974

Abbreviations: Coefficient: linear regression coefficient; FDR: false discovery rate p value, with those in bold type significant at α ≤ 0.05.

**Figure 2:**
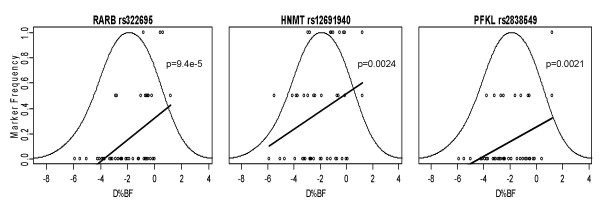
Physiogenomic representation of the most significant genetic associations found in the low fat diet group. Individual patient genotypes (*circles*) of each SNP are overlaid on the distribution of Δ%BF (*thin line*). Each circle represents a patient, with the horizontal axis specifying the Δ%BF, and the vertical axis the carrier status for the minor allele: bottom, non-carriers; middle, single-carriers; top, double-carriers. A LOESS fit of the allele frequency (*thick line*) as a function of Δ%BF is shown. The ordinate is labeled for the marker frequency (*thick line*) of the SNP denoted at the top of each panel. The ordinate scale is the same in all three panels. The ordinate scales for the genotypes (*circles*) and Δ%BF distribution (*thin line*) are not shown. The abscissa is labeled for Δ%BF in each panel. The abscissa scale is the same in all three panels and applies identically to marker frequency, genotypes, and Δ%BF distribution.

Figure 2 shows the top three markers (according to p value) related to LF response. The first panel shows the LOESS curve for SNP rs322695 of the *RARB *gene. The frequency of the minor allele increases as the Δ%BF response to the LF diet becomes less pronounced (i.e., no loss of relative fat mass). The minor allele is completely absent among subjects with the largest decreases in %BF, and the frequency is <10% among subjects whose decrease in %BF exceeded 1%. This finding indicates a strong association between the *RARB *marker and response to LF. Similar patterns are seen for *HNMT *rs3100722 and *PFKL *SNP rs2838549. The SNPs, *RARB *SNP rs322695, *HNMT *SNP rs3100722, and *PFKL *SNP rs2838549, are considered "torpid" markers for responsiveness to the LF diet.

**Figure 3:**
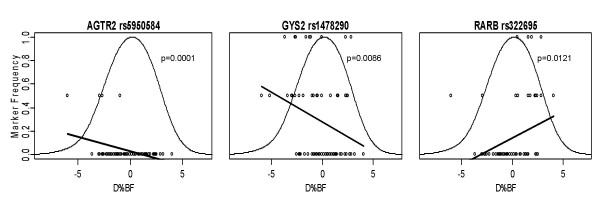
Physiogenomic representation of the most significant genetic associations found in the low carbohydrate group. See Figure 2 legend for details regarding individual patient genotypes (*circles*), the distribution of Δ%BF (*thin line*), and the LOESS fit of the allele frequency (*thick line*) as a function of Δ%BF.

Figure 3 shows the top three markers associated with Δ%BF through LC. The first panel in Figure 3 shows the LOESS curve for SNP rs5950584 of the *AGTR2 *gene. The frequency of the minor allele is 30% in the Δ%BF range that is less than -5%, and the minor allele is completely absent among subjects with subtle change in Δ%BF (i.e., no loss of relative fat). A similar pattern is seen with the *GYS2 *SNP rs1478290. These two SNPs are considered reactive markers. The third panel shows the *RARB *SNP rs322695 response. The minor allele shows a higher frequency in the subject with little response in Δ%BF, indicating that the *RARB *SNP rs322695 is a torpid marker of responsiveness to LC diet also.

## Discussion

The present study shows that genetic associations with changes in Δ%BF established for genes in pathways encompassing food intake, energy homeostasis, and adipocyte regulation occur in part through common pathways, and in part through different pathways that may differentiate low fat (LF) and low carbohydrate (LC) restriction. For both diets, Δ%BF profiles assessed using DXA were affected by an intergenic SNP upstream of *RARB *(rs322695), suggesting that *RARB *is involved in a fat loss pathway common to both diets. In contrast, strong diet-specific associations were also found for a promoter region SNP in *AGTR2 *(rs5950584) through LC and *PFKL *through LF, suggesting that there are separate mechanisms for fat loss under the LF and LC diets.

### Physiogenomic associations common to both LF and LC

We observed a significant relationship between the *RARB *SNP rs322695 and change in relative fat to *both *diets after accounting for nongenetic factors. The relationship was significant after correction for multiple testing in mediation of the LF Δ%BF profiles (p < 0.005). This observation is noteworthy in light of the putative role of the retinoic acid system in insulin resistance [60]. The *RARB *SNP rs322695 is found 100 kb upstream and ~600 kb from the adjacent *THR *(thyroid hormone receptor) gene. We have attributed this SNP to *RARB *because it is the next gene downstream, and regulatory elements such as enhancers have been found several hundred kb from their transcription start sites. However, it cannot be excluded that the SNP effect may be unrelated to *RARB *protein expression, and rather involve an as yet uncharacterized protein or non-coding RNA [61]. We are unaware of other polymorphisms in the region or near *RARB *that are related to body weight or fat regulation in humans and believe this finding to be novel.

The widespread genomic distribution of retinoic acid response elements (*RARE*) suggests participation in many physiological pathways [62-64]. Recently Morgan et al. [40] described a neural hypothesis for regulation of annual cycles of body fattening in animals in which changes in the expression of *RAR *in the arcuate nucleus of the brain were noted. Vitamin A deficiency preferentially decreases hepatic *RARB *expression [65], and regulates hepatic glucose metabolic enzymes [66]. Given that hepatic enzyme energy regulation occurs in part through *RARB *signaling, the present finding allows the hypothesis that the rs322695 variant affects such regulation.

### Physiogenomic associations specific to LF

In addition to the *RARB *gene, LF Δ%BF profiles had relationships through *PFKL *and one SNP in *HNMT *not found with LC. The *HNMT *SNP rs3100722, found at intron 2, and the *PFKL *SNP rs2838549, located in intron 8, showed significant relationships (p < 0.041) after correction for multiple tests) second in strength only to the *RARB *gene. The *HNMT *inhibitor metoprine suppresses feeding in mice [67]. The present finding enables us to hypothesize a role for *HNMT *in the regulation of food intake. Hepatic phosphofructokinase, a key regulatory enzyme for glycolysis encoded by *PFKL*, is responsive to macronutrient changes [68] and is regulated by Vitamin A [66]. Genetic loci near *PFKL *have been associated with bipolar affective disorder [69], but to our knowledge, no SNPs are known to modulate diet response.

### Physiogenomic associations specific to LC

The strongest association (p < 0.003, after correction for multiple tests) was found in the LC group with SNP rs5950584 in the angiotensin II receptor, type 2 (*AGTR2*). This SNP is found ~4.5 kb upstream in the promoter region. The *AGTR2 *gene has a metabolic role that is in contrast to the vascular role of the angiotensin II type 1 receptor (*AGTR1 *gene), which we previously found linked to statin-associated elevations in serum creatine kinase [17]. Humans and mice express *AGTR2 *in muscle and adipose tissue, and evidence supports the existence of a functional renin angiotensin II system within adipose tissue [70]. Mice lacking the *AGTR2 *receptor are resistant to the adipocyte hypertrophy and muscle cell insulin resistance induced by high fat, hypercaloric feeding [71]. *AGTR2*-dependent angiotensin II signaling thus could account for LC unresponsiveness [71]. *AGTR2 *polymorphisms were reported to modulate left ventricular mass, through the polymorphism known as G1675A (rs1403543) in a European population [72], and the T-A combination derived from G/T rs5193 and G/A rs5194 SNPs in a Cantonese population [73].

In the present study, the *AGTR2 *SNP rs5950584 serves as a reactive marker for the LC diet response. The *AGTR2 *gene is X-linked, thus men can have only one of either the T (major) or the G (minor) allele, while women may have zero, one, or two copies of either allele. Men lost significantly more fat than women, which together with the X-linked nature of the gene might lead to a false positive result. However, gender was highly significant in our covariate model, and the association test was performed adjusting for it, which excludes such a confounding effect.

Another association was found between LC response and the SNP rs1478290 in the *GYS2 *gene. The *GYS2 *product is glycogen synthase 2, a key regulator of hepatic glucose storage that is increased by feeding [74]. The *GYS2 *SNP rs1478290 is found in the promoter region. We previously found a different *GYS2 *SNP, rs2306179, located in intron 5, to be associated with body weight loss in response to LC diet [5]. The present study, conducted in a larger number of subjects, found also that a variant in the *GYS2 *gene is a response marker for the change in relative fat induced by LC.

### Differentiation of LF and LC diet responses

The number of significant associations overall suggests a complex system through which regulation of the %BF phenotype is accomplished. Our list of genes, which is comparable to other studies to date [15], is exploratory, not comprehensive, and is representative of food intake, energy homeostasis, and adipocyte regulation. Virtually none of the genes in the present study were examined by previous diet studies [13,15]. Nevertheless, the present results showed a larger number of gene associations with Δ%BF profiles in relation to the LF diet compared to the LC diet. Based simply on the numbers of genes found to be significantly associated, we infer that the low fat diet utilizes a greater number of signal inputs into the regulation of relative body fat compared to the low carbohydrate diet. Even after the most stringent correction for multiple testing, RARB remained significantly associated with LF Δ%BF and AGTR2 remained associated with LC Δ%BF.

Our findings run counter to those of a recent study [15] that reported no significant SNP associations with changes in BMI following carbohydrate or fat restriction despite using large cohorts. Methodological differences between the studies are worth noting. The present study used DXA to precisely assess Δ%BF as opposed to relying on BMI. Second, different genes were studied. Here, the selection of genes was designed to reflect Δ%BF phenotype variability. In contrast, the SNPs examined by Sorenson et al. [15] were those demonstrated in previous reports to affect obesity and not diet response *per se*. Macronutrient compositions of the intervention diets were quite dichotomous in the present study, especially for carbohydrate (~12% and 57% of total energy as carbohydrate in the LC and LF arms, respectively, in the present study; *vs*. 42% and 57%, respectively, in the study of Sorenson et al. [15]). We believe a greater imbalance in the macronutrient combination increases the strength of the dietary stimuli, which accentuates differences in the physiological signal intensities associated with each diet, increasing the chances for detection of physiogenomic relationships.

### Clinical implication

The prevention and treatment of obesity could be more efficient if dietary recommendations were carried out based on knowledge of innate individual characteristics that are genetically predictable. Moreover, adherence to dietary advice may increase when the advice is personalized. DNA-guided regimens allow healthcare providers to tailor therapies based on individual patient characteristics, rather than the average diet responses applied in longitudinal studies [3,75]. A dietary treatment with a higher average chance for success, for example, may be desirable for those patients who inherited an ensemble of DNA markers with the most responsive factors and the least torpid factors for it. We interpret the coefficients associated with each significant gene marker in Table 4 as either having responsive or torpid effects to facilitate fat loss. The ability to match a diet regimen's genetic "contour" with the DNA profile of an individual patient marks the beginning of high-resolution personalized nutritional medicine for the treatment and prevention of obesity.

### Limitations

The number of subjects studied, the list of genes analyzed, and in some cases the frequency of the minor allele in SNPs of interest, were relatively small. Thus the results will need to be validated before clinical application. Nevertheless, the *RARB *and *AGTR2 *results are clearly significant even when fully and most conservatively corrected for multiple comparisons. The p-values were confirmed by non-parametric permutation analysis, excluding non-normal distribution as a source of false positives. In addition, the RARB result was found in the LF group and independently confirmed in the LC group. Although we assessed only a small sample of SNPs in the genome, this study included 53 SNPs from 28 genes representing a larger ensemble of genetic probes than other published diet studies. The data for the present study were compiled from a series of studies in whom the majority of subjects were of European ancestry. We lack power to detect ethnogeographic associations or confounding effects. We did not consider men and women separately but accounted for the effect of gender through its use as a covariate. Future studies investigating a sex difference might be useful. Finally, both diets were hypocaloric. It is possible that the genes shown to exert their effects through both diets, actually do so through the commonality of caloric restriction.

## Authors' contributions

RLS drafted the manuscript. JSV conceived of the study, designed and executed many of the diet restriction studies that supplied DNA, and helped draft the manuscript. AW participated in the design of the study, oversaw the genetic analyses, and performed the statistical analysis. MK performed the molecular genetic analyses. MLF participated in the design of the study and conducted some of the diet restriction studies that supplied DNA samples. WK participated in the design and coordination of diet restriction studies that supplied DNA and phenotype information. GR participated in the study design, provided oversight for genetic analyses and helped to draft the manuscript. All authors read and approved the final manuscript.
